# Surface Plasmon Resonance Aptasensors: Emerging Design and Deployment Landscape

**DOI:** 10.3390/bios15060359

**Published:** 2025-06-04

**Authors:** Fahd Khalid-Salako, Hasan Kurt, Meral Yüce

**Affiliations:** Sabanci University Nanotechnology Research and Application Center, 34956 Istanbul, Türkiye; fahd.khalidsalako@sabanciuniv.edu (F.K.-S.); hasankurt@sabanciuniv.edu (H.K.)

**Keywords:** surface plasmon resonance, aptamers, SELEX, biosensors, ligands, drug discovery

## Abstract

SPR biosensors operate on the principle of evanescent wave propagation at metal–dielectric interfaces in total internal reflection conditions, with consequent photonic energy attenuation. This plasmonic excitation occurs in specific conditions of incident light wavelength, angle, and the dielectric refractive index. This principle has been the basis for SPR-based biosensor setups wherein mass/concentration-induced changes in the refractive indices of dielectric media reflect as plasmonic resonance condition changes quantitatively reported as arbitrary response units. SPR biosensors operating on this conceptual framework have been designed to study biomolecular interactions with real-time readout and in label-free setups, providing key kinetic characterization that has been valuable in various applications. SPR biosensors often feature antibodies as target affinity probes. Notably, the operational challenges encountered with antibodies have led to the development of aptamers—oligonucleotide biomolecules rationally designed to adopt tertiary structures, enabling high affinity and specific binding to a wide range of targets. Aptamers have been extensively adopted in SPR biosensor setups with promising clinical and industrial prospects. In this paper, we explore the growing literature on SPR setups featuring aptamers, specifically providing expert commentary on the current state and future implications of these SPR aptasensors for drug discovery as well as disease diagnosis and monitoring.

## 1. Introduction

Recent advancements in biosensor materials and the integration of artificial intelligence in data acquisition and monitoring have led to multiplexed SPR biosensors developed, for example, to diagnose COVID-19, other infectious diseases, and detect small molecule targets in complex biological media with high sensitivity and clinical performance [1]. Crucially, SPR biosensors have also been extensively adopted in the pharmaceutical industry. In addition to label-free detection, they provide real-time binding data, enabling the in-depth characterization of the kinetics underpinning biomolecular interactions. This capability of SPR biosensors has led to their integration into drug discovery and development workflows to better identify promising drug candidates and evaluate pathophysiologically relevant binding events.

Antibodies have been the biorecognition molecules of choice in biosensors due to their high specificity and affinity for a wide range of analytes. However, they are disadvantaged in their instability and strict production and handling requirements that drive up costs of production and limit their use at scale. Alternative probe molecules have been developed to address the challenges with antibodies, the most prominent of which are aptamers, small oligonucleotides or peptides with structural flexibilities, adopting tertiary structures that impart high affinity and specific binding to a wide range of targets like proteins, peptides, antibodies, small molecules, and toxins, among others [2].

Aptamers are a relatively recent technology developed via an in vitro iterative selection process less than 40 years ago [3]. Structurally, they are peptide or oligonucleotide sequences comprising 20 to 200 nucleotides. They are a quintessential outcome of experimental science; while the theory underlying their high-affinity binding to bio-targets remains a subject for active, ongoing research, they have already been utilized in several industrial and scientific applications, having found use in biomarker research, molecular diagnostics and disease monitoring, environmental monitoring, materials science, and clinical biotherapy applications [2,4,5]. The binding of aptamers to their targets has been hypothesized to be mediated by complex 3D structural conformations of the aptamer molecules, snugly fitting the targets to form complexes stabilized by combinations of hydrogen bonds, electrostatic, aromatic interactions, and van der Waals forces, among others [6].

The advantages of aptamers over protein- and antibody-based affinity probes arise from their relative simplicity, ease of production at scale, thermal and chemical stability, and flexibility and reversibility of structural deformations, making them less immunogenic, cost-effective, easy to handle, more amenable to chemical functionalizations, and less prone to batch-to-batch variations, compared to their biologically produced counterparts [4,7]. These functional merits have driven their wide adoption in biosensor research and translational adoption. Aptamers have become monumental in the biomedical sciences and clinically, for both diagnostic and therapeutic purposes. An aptamer-based therapeutic received regulatory approval for treating age-related macular degeneration 20 years ago, becoming the first clinically approved aptamer therapeutic [8].

While the potential of aptamer-based biosensors is undeniable, there appears to be minimal instances of SPR aptasensors making it to translational adoption, compared to other aptasensor setups. In this review, we examine the literature on aptasensors with SPR signal transduction modality, providing a comprehensive overview of the theoretical basis and applications of SPR biosensors, as well as design considerations of SPR aptasensors, to facilitate a general understanding of the contemporary SPR aptasensor literature landscape. We also present the translational perspective of SPR aptasensors, discussing current challenges, limitations, and future perspectives.

## 2. SPR Biosensors: Theoretical Basis and Applications

### 2.1. The Principle of Surface Plasmon Resonance

The surface plasmon resonance (SPR) phenomenon first emerged as an observable optical phenomenon in the early twentieth century, with experimental demonstrations of inexplicable intensity patterns in polarized light reflected from metal-backed diffraction gratings, termed Wood’s anomalous diffraction gratings. Theoretical studies that followed this discovery culminated in the postulation of polarized quasi-stationary waves existing close to the boundaries of metallic surfaces, excitable by incident irradiation under critical conditions [9]. The excitation of these surface plasma oscillations or polaritons led to interference patterns in reflected light and was dependent on optoelectronic events just beyond the boundaries of the metal surfaces. This principle was operationalized in the mid-70s in the development of ‘surface polariton probes’ of surfaces and interfaces for the characterization of metallic films and coatings [10]. Later, one of the first applications of SPR in biosensing was demonstrated by applied physicists at the Linköping Institute of Technology, Sweden, in 1983 when they experimentally demonstrated SPR shifts due to human immunoglobulin in aqueous solution, flowing through a 2 mL cavity with one of its walls being a silver film-coated glass prism [11]. Several SPR biosensors have since been developed, utilizing surface plasmons as sensitive probes of dielectric media interfacing thin metallic surfaces, and scientific interest in SPR biosensors continues to rise.

Studies on the theoretical basis for the SPR phenomenon have produced detailed frameworks of the resonance condition’s dependencies. One of the most extensively cited mathematical models for the SPR phenomenon is Fresnel’s equations, which model SPR as a complex physical resonance event dependent on an interplay of incident light wave and material properties of the setup’s components [12].

P-polarized light incident on a metal surface propagates with a wave-vector component, perpendicular to the metal-dielectric interface k_z_ defined by the following [12]:

(1) $$k_{z}=\sqrt{\frac{\varepsilon_{m}*\omega^{2}}{c^{2}}-k_{x}^{2}}$$

where ω and c, respectively, represent the photonic wave angular frequency and speed of light; ε_m_ denotes the dielectric constant of the metal; and k_x_ is the wave-vector component parallel to the metal–dielectric interface, defined by [12]

(2) $$k_{x}=\frac{\omega \sqrt{\varepsilon_{g}}}{c}sin\theta$$

where ε_g_ is the dielectric constant of the material on the side of the metal adjacent to the incident irradiation (presumably a glass prism), and θ is the angle of incident light in radians. Equations (1) and (2) demonstrate the dependence of incident light wave properties, not only on the incident angle (θ) and wavelength/frequency (ω), but also on the properties (dielectric constants) of the metal itself and the material on the side of the metal, irradiated by light. This provides an avenue to optimize SPR biosensor performance by precisely tuning their designs and component materials.

In the hypothetical absence of a glass prism interfacing with the metal sheet on the other side, surface plasmons at a metal–dielectric medium interface would predictably oscillate with a calculated wavenumber, k_0_ [12]:

(3) $$k_{0}=\frac{\omega }{c}\sqrt{\frac{\varepsilon_{d}*\varepsilon_{m}}{\varepsilon_{d}+\varepsilon_{m}}}$$

where ω here represents the angular frequency of plasmonic oscillation and ε_d_ denotes the dielectric constant of the dielectric/sensing medium. In Equation (3), the dielectric constant of the sensing medium comes to fore, showing how changes to the sensing medium such as refractory index changes, which affect its dielectric constants, might affect surface plasmons. This demonstrates the mechanism by which binding events in SPR biosensors are detected as resonance shifts.

In a theoretical model described by K. Kurihara et al. (and widely adopted in the literature to date), building on the simple Kretschman SPR configuration, plasmonic excitation and consequent photonic energy attenuation occur in the following condition [12]:

k_x_ = k_0_ (4)

Representing the resonance of parallel components of both photonic and plasmonic waves across a sufficiently thin metal film, which occurs only in the presence of a glass prism or similar optical material, introducing a third dielectric constant (ε_g_), to account for negative ε_m_, and imparting a perturbation in the surface plasmonic wave number such as that in resonance conditions, the parallel component of their oscillation wave number (k_sp_) is given by the following [12]:

(5) $$k_{sp}=k_{0}+k^{*}$$

where k*, the prism-mediated perturbation, is mathematically dependent on amplitude reflectance at the prism–metal interface at resonance, ε_m_, ε_d_, the thickness of the metal, and the wavelength (λ) of incident light [11,12]. While slightly different mathematical models exist to accommodate the peculiarities of other SPR configurations apart from Kretschmann’s, they share similarities in the major resonance condition dependencies, which are exploited through instrumentation design to institute a highly sensitive SPR shift response to changes in the sensing dielectric medium.

In essence, surface plasmon resonance is a phenomenon that is characterized by light transferring energy to and exciting waves at the boundaries of metallic materials. This excitation occurs in very specific conditions (the resonance conditions) and results in measurable changes in the properties of the light reflected from the metal surface, such as its intensity and phase. Detecting these changes and determining the resonance conditions provides valuable information on the optical properties of the interaction medium, and it forms the basis of SPR biosensors. A technical discussion of the factors governing the resonance event follows.

Crucially, incident light in an SPR setup is required to be p-polarized, such that the electric field component is perpendicular to the metal surface, allowing optimal interactions with surface polaritons. Additionally, the optical properties of the metal play an important role in the SPR event. Conventionally, gold (Au) and silver (Ag) have been used most extensively in SPR systems, owing to their electronic properties and biological compatibility as noble metals [13,14]. However, Au and Ag have limitations of scarcity, difficulty in bandgap tuning, and control of opto-electronic features for plasmonic applications. As a result, several other metallic and semi-conductor plasmonic material systems have been explored, like metal oxides, carbon-based nanomaterials, non-metallic materials, two-dimensional nanomaterials, and importantly, transition-metal nitrides, especially when tunability, thermal stability, and scalability are desired for sensing and other applications [14,15]. It is important to note that the electronic properties of these different material systems manifest in their permittivity/dielectric constant and have significant impacts on the SPR conditions, as well as the wave properties of the generated surface plasmons, like probing depth and field confinement [13].

In addition to the wave properties of the incident light and optoelectronic properties of the plasmonic metal/material, certain experimental conditions also influence the plasmonic resonance event and conditions. The angle of light incidence (θ) is one such crucial condition, as is demonstrated in the mathematical model described (Equations (2)–(4)). The parallel wave-vector of incident light depends on θ (Equation (2)), introducing it into the eventual resonance equation. Importantly, several SPR instruments in different configurations are designed to vary incidence angles during measurement, such that a shift in the angle at which resonance is observed is recorded in an arbitrary unit as a real-time measure of the binding event in the sensing medium. The resonance angle was, in fact, the major binding signal transduction principle of the SPR biosensor developed by Liedeberg and colleagues when they demonstrated the first SPR application in biosensing [11]. Another SPR dependency commonly exploited in SPR biosensors—similarly to the incident angle—is the incident light wavelength. We see this in the k_x_ term of the resonance condition (Equation (4)), with ω, denoting the angular frequency of the incident light wave, which is inversely proportional to the wavelength and provides a mathematical description of the photonic momentum/energy state. SPR biosensors designed to detect resonance wavelength shifts (typically at constant incident angles) are said to conduct wavelength interrogation [16].

Plasmonic resonance is also dependent on the optical properties of the sensing medium adjacent to the metal surface. This is the factor that provides the biosensing capacity and sensitivity of the physical phenomenon. The refractive index and permittivity of the medium significantly influence SPR conditions, such that shifts in resonance angle or wavelength are highly sensitive to mass-induced changes in the sensing medium, often occurring consequent to an analyte binding to an immobilized affinity probe. Importantly, the wave properties of the surface plasmons, peculiar to the plasmonic materials, determine the sensitivity of SPR biosensing, due to the depth of plasmonic penetration into the sensing medium (probing depth) and field confinement which determines signal resolution through plasmonic localization along the interface [13].

Taken together, SPR dependencies are exploited through precisely engineered biosensor systems to obtain rich data on biomolecular interactions for a plethora of purposes. Steady-state affinity data rivaling legacy immunoassays can be obtained, with the added advantage of label-free experimental workflows. Dynamic time-plot series (sensorgrams) that uncover interaction kinetics can also be obtained from SPR biosensors. Recent advancements in materials engineering, functionalization, and instrument design have opened up new notable possibilities to explore biochemical interactions at a deeper level with SPR biosensors. These reported advancements include multi-wavelength biosensing [16], phase change interrogation [17], SPR imaging on nanopatterned sensor substrates [18], and machine learning-powered big data acquisition and processing possibilities, among others [1]. Requirements for certain data types and broader biosensing needs and circumstances have given rise to various SPR biosensor configurations with peculiar characteristics. These form the crux of the discussion in the next section.

### 2.2. SPR Configurations

In SPR systems, momentum and energy remain conserved, and the photonic attenuation observed at resonance results in plasmonic excitation. Notably, photons naturally have momenta that are insufficient to excite surface plasmons. To address this, some configurations have been developed to increase the momentum of photons and provide the possibility of resonance at the right conditions. These include prism-coupled and diffraction grating-based configurations, as well as fiber optics and waveguide-based setups [16]. Additional biosensing data requirements as well as advanced applications of SPR have also given rise to other configurations like SPR imaging (SPRi), localized SPR (LSPR), and electrohydrodynamic microfluidics for improved sample handling and high-throughput screening.

**Figure 1:**
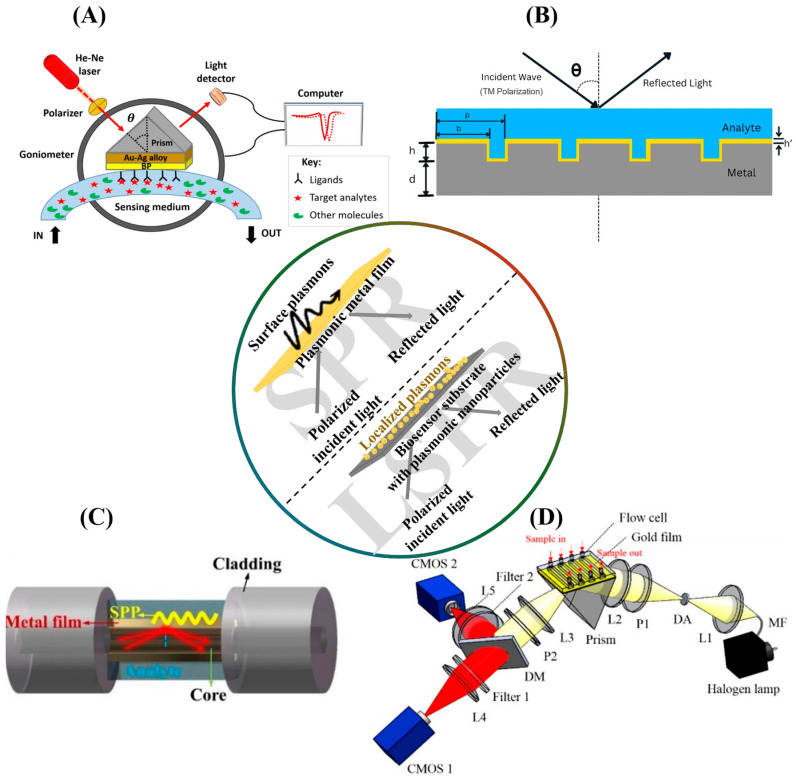
An illustration of the major SPR biosensor configurations. The inset diagram demonstrates the general principle of SPR and LSPR, juxtaposing film-confined evanescent waves in SPR with localized plasmons in LSPR upon polarized light irradiation. (**A**) A schematic of the conventional Kretschmann SPR configuration. Reproduced with permission from ref. [34]. Copyright 2025 Elsevier B.V. (**B**) A typical diffraction grating-based SPR setup. p, grating period; b, grating bar width; h, grating thickness; h’, plasmonic metal coating thickness; d, substrate thickness; θ, incident angle (slight modifications made for editorial and formatting purposes) [19]. (**C**) A diagrammatic sketch of a transmissive fiber optic SPR sensor. SPP, Surface plasmon polaritons. Reproduced with permission from ref. [20]. Copyright 2022, Wiley-VCH GmbH. (**D**) An SPR imaging (SPRi) system based on the Kretschmann configuration. L1–L5, lenses; DA, diaphragm aperture; MF, multimode fiber; DM, dichroic mirror; P1 and P2, polarizers [35].

Prism-coupled SPR setups were the first configurations in which SPR was experimentally applied for chemical characterization purposes and remain the most widely used configuration in SPR biosensors. In a prism-coupled SPR biosensor, incident light typically passes through a prism made of a high-refractive-index material, where total internal reflection occurs, coupling the incident light wave to plasmons at the surface of a metal film adjacent to the prism (Figure 1A). This is the general setup for the Kretschmann and Otto SPR configurations. In the Kretschmann configuration, however, the prism is typically in contact with the metal film, while Otto configurations utilize a small gap containing air or other dielectric materials to separate the prism from the metal surface. This provides better flexibility and tunability in photon–plasmon interactions through precise instrumentation design [13].

While prism-coupled SPR represents the most widely used configuration and the setup on which legacy mathematical modeling of SPR biosensors is based, it is noteworthy that Wood’s anomalous behavior of light waves, the first experimental observation of plasmonic resonance that eventually gave rise to SPR biosensors, was observed in metal-backed diffraction gratings. Instead of prisms, diffraction grating-based SPR biosensors use diffraction gratings (periodic patterns on the metallic surface), with slits that act as point wave sources for the diffracted light wave at varying angles, resulting in photonic momentum reaching levels capable of plasmonic excitation. Diffraction grating-based SPR configurations are advantageous over prism-coupled SPR biosensors. They are more easily adjustable through the modification of grating parameters (Figure 1B). Additionally, diffraction grating-based SPR setups are more compact and amenable to miniaturization, enabling their use in point-of-care testing and portable biosensor devices while being more cost-effective and economically viable at scale [19].

Advancements in the fabrication and functionalization of materials have enabled further miniaturization and operationalization of SPR biosensors based on innovative optical waveguides and fibers. Optical fibers are typically made of two materials with different refractive indices, which comprise the core and cladding. The interaction of incident light within this biphasic optical environment ensures perpetual total internal reflection along the length of the fiber at incidence angles beyond the critical angle. Optical fiber-based SPR setups exploit this by incorporating a metal–sensing medium interface in an unclad area of the fiber, effectively generating plasmonic probes from broadband incident light for the wavelength interrogation of the sensing medium (Figure 1C). These systems overcome the bulkiness of prism-coupled setups, providing compactness, the possibility of integration within multiplexed sensing systems, and remote monitoring functionalities [20]. In addition to fibers, optical waveguides have also been functionalized with plasmonic metal films for waveguide-based SPR sensing, providing additional possibilities for incorporating other sensing principles within the same physical setup for multimodal biosensing [21].

SPR biosensors in their base forms are typically label-free setups that detect mass-induced changes in the dielectric medium due to analyte binding, reflecting in a proportional shift in resonance angle or wavelength. By implication, the sensitivity of the biosensor depends on the physicochemical properties of the analyte and its affinity probe, especially their relative molecular weights and size. This limitation on the sensitivity of SPR biosensors has resulted in the development of localized SPR (LSPR) and nanoconstruct-based sensor surfaces, which provide sensitivity improvements without the need for sample labeling for signal amplification. Surface plasmons typically propagate along the metal film–dielectric interfaces, generating evanescent waves that probe the dielectric media. However, in nanosized plasmonic particles (often smaller than or the same size as the incident light wavelength), the plasmonic fields are confined to the surfaces of the nanoparticles and are easily excitable (Figure 1). This plasmonic localization improves the biosensor’s sensitivity and performance, with resonance shifts highly responsive to changes close to the nanoparticles [22]. LSPR biosensor setups with different coupling configurations have been developed for this reason. Importantly, recent studies have demonstrated prism-coupled and glass-coupled LSPR setups, fabricated by coating purified glass slides with gold nanoparticles, nanorods, and nanodisks, among other nanoconstructs, leveraging the high refractive index, transparency, and optical properties of glass to enable localized plasmon generation at nano-surfaces [23,24,25,26]. As nanofabrication technologies develop, challenges associated with LSPR biosensors, like high radiation losses, are surmounted with precisely controlled nanoparticles of pre-determined morphologies, arrangement, and distribution on sensor substrates [27,28]. The inherent compactness of LSPR systems also drives their clinical adoption, and one such LSPR biosensor was recently developed for the point-of-care diagnosis of COVID-19 [29].

In SPR imaging (SPRi), a heterogeneously functionalized sensor surface is typically irradiated with defocused incident light, with the reflected light recorded by a charge-coupled device (CCD) camera, producing a two-dimensional map of SPR activity on the entire sensor surface, with high spatiotemporal resolution (Figure 1D). SPRi setups often utilize LSPR, exploiting the high electronic localization to more effectively capture SPR at each pixel. SPRi has notably been applied in different fields of research, such as monitoring biochemical activities of microorganisms and cells, as well as environmental monitoring and clinical diagnostics [30,31]. In addition to SPRi, some other innovative SPR-based biosensor setups have been designed to address the increasing demand for compactness, high-throughput screening, and smart biosensors. Some of these approaches include miniaturized SPR chips for use with smartphones [32] and electrohydrodynamic microfluidic blocks for more efficient sample handling, miniaturization, and high-throughput screening [33], among others. Ongoing research continually builds on these advancements to improve the sensitivity, efficiency, and scalability of SPR biosensors through key design, material functionalization, data acquisition, and crunching approaches.

### 2.3. SPR Applications

SPR biosensors have found broad use in research and development, for drug discovery, development, and lead optimization purposes. They have also been used for biomarker identification and characterization, disease modeling, and clinical diagnostic and disease monitoring purposes. Industrially, SPR biosensors are used for drug biosimilarity analyses and material characterization. They also play important roles in environmental monitoring applications. The versatility of SPR biosensors is underpinned by the mass-mediated signal transduction modality, which eliminates the need for labeling steps that often compromise test analytes. Additionally, biomolecules often perform their pathophysiological roles through highly specific interactions with conjugate pair molecules; this is the principle that underlies many immunoassays. Advancements in chemical biology and the wide variety of immobilization strategies for these conjugate pair molecules on SPR sensor surfaces have also driven their biomedical applications, establishing the technology as mainstay in clinical and pharmaceutical research industries.

In pharmaceutical research, the safety and efficacy of novel therapeutics can be estimated by a thorough assessment of their binding dynamics within drug–target, antigen–antibody, and protein–receptor complexes. In their simplest form, SPR bioassays can provide binding kinetics data, representing complex formation and decay rate constants, as well as steady-state affinity values. Through advanced experimental designs, SPR biosensors can also be used to evaluate the thermodynamic stability of complexes, obtain stepwise rate constants of a multi-step process for a more elaborate representation of mechanisms of action, and conduct high-throughput drug screening, typically following in silico experiments to validate computational findings and quickly compare multiple lead drug compounds [13]. In the same vein, SPR biosensors have also been used to conduct the biosimilarity screening of new biologics, based on their functional similarity with innovator products, demonstrable by their target binding characteristics [36].

In clinical use, SPR biosensors offer immense capabilities in the characterization of protein and nucleic acid biomarkers that have theoretically been demonstrated to contribute to the pathogenesis, pathophysiology, and progression of infectious diseases and other chronic diseases like cancers, autoimmunity, and neurodegeneration. Additionally, the compact designs of advanced SPR configurations contribute to their potential for clinical deployment to diagnose and monitor diseases, through the highly specific detection and accurate quantification of biomarkers. SPR biosensors can be functionalized with innovative surface preparation strategies that make them specific and selective for target analytes, making them capable of reliably detecting key biomarkers at low levels within complex biological media and clinical samples like blood [37,38,39], synovial fluid [40,41], saliva [42], and urine [1].

**Figure 2:**
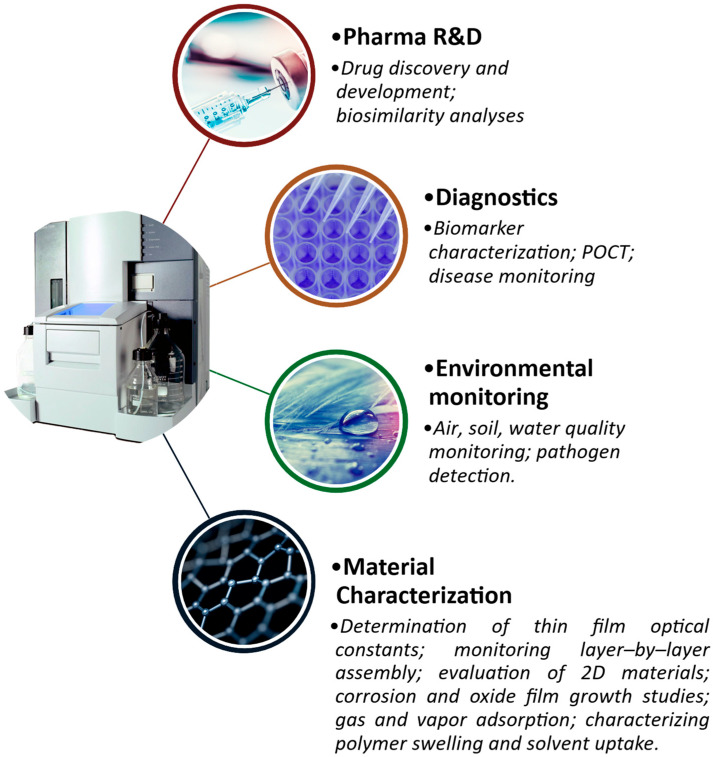
SPR biosensors have found widespread use in pharmaceutical research and development, clinical diagnostics, environmental monitoring, and the characterization of thin films’ structural and optical properties.

Beyond industrial and clinical biomedical applications, SPR biosensors have also demonstrated usefulness in environmental monitoring. The versatility of SPR biosensors makes them amenable to surface functionalization with a wide range of affinity probes capable of detecting pollutants in environmental samples, as well as agricultural produce. SPR biosensors can similarly be functionalized to detect volatile and air-borne chemical markers of air quality and environmental quality monitoring. In the agricultural industry, common pathogens can be detected and quantified in soil, water, the air, and living tissue to contain communicable diseases in plants and livestock [43]. In addition to characterizing binding interactions between conjugate-pair molecules, SPR has also been applied in material characterization to obtain the optical characteristics of thin films, providing data on the complex refractive indices and thickness of thin films deposited on plasmonic metal surfaces [44]. An overview of the applications of SPR biosensors in various fields is presented in Figure 2.

## 3. Design Considerations in SPR Aptasensors

### 3.1. Aptamer Selection and Properties

Aptamers are small oligonucleotide or peptide biomolecules typically produced in vitro, which bind biochemical targets with high affinity and specificity, due to their structural flexibility and intrinsic tertiary folding behaviors. One of the first attempts at the molecular evolution of oligonucleotides appeared in the literature in 1988, when scientists at the Salk Institute for Biological Studies, USA, reported an iterative workflow for the mutation, selection, and amplification of RNA for catalytic purposes [45]. Less than a year after this study, Kinzler and Vogelstein reported a PCR-based amplification of DNA sequences with affinity for gene regulatory proteins [46], laying the groundwork for Ellington and Szostak, who generated RNA subpopulations from a combinatorial nucleic acid library, with high affinity for organic dyes [3], and Bock et al., who, for the first time, generated single-stranded DNA aptamers with high affinity for biomolecules with which they have no known physiological interactions [47]. This was a monumental leap in aptamer technology as it ascertained the versatility of aptamers, demonstrating that aptamers can be synthesized to bind a wide variety of molecules, even those with which they do not interact in vivo. Aptamers have since been produced and reported in the literature with affinity for a wide range of targets like heavy metals, toxins, proteins, whole cells, and small molecules, among others [4]. Ellington and Bock are widely acknowledged as pioneers of aptamer production, together with Tuerk and Gold, who, coincidentally, at the same time as Ellington and Szostak, iteratively selected RNA molecules to bind a bacteriophage polymerase [8,48]. This iterative selection technique is termed Systematic Evolution of Ligands by Exponential Enrichment (SELEX). While different modified SELEX techniques have been developed, they all follow a simple workflow that starts with a random library and iterates through incubation, elution, and amplification steps, until a high-specificity and -affinity subpopulation is reached and sequenced, enabling reproducible chemical synthesis [4].

SELEX technologies have evolved through more efficient incubation and elution processes. Advancements in gene sequencing and computational methods have also further increased the scalability and efficiency of SELEX methodologies. Some of the conventional SELEX techniques discussed in the previous literature include magnetic bead-based SELEX, capillary electrophoretic SELEX, and whole-cell SELEX [4]. Magnetic bead SELEX methods are simple workflows in which magnetic beads are coated and functionalized with target analytes using different covalent and non-covalent surface preparation techniques. The analyte-coated magnetic beads are incubated with the starting aptamer library and can be easily magnetically eluted to obtain bound aptamer subpopulation in each iteration of the process. In capillary electrophoresis SELEX, the analytes are incubated with aptamer library in a label-free liquid medium, after which analyte-bound aptamers are separated by capillary electrophoresis, purified, and amplified. Whole-cell SELEX generates aptamers for whole cells, as opposed to the well-defined and purified target analytes in other techniques. Whole-cell SELEX fully epitomizes the experimental approach of SELEX technology, incubating whole cells about whose surface proteins very little is known, with the aptamer library in a free medium, and separating cell-bound aptamers by centrifugation. Whole-cell SELEX has been applied principally in producing aptamers against microorganisms. In an advancement of the whole-cell SELEX approach, cells can be engineered to overexpress pre-determined surface proteins for incubation close to their natural conformations, using knockdown cells as negative control to improve the specificity of the generated aptamers [4]. Flow cell SELEX involves the incubation and elution of non-binding aptamers on an SPR chip with a microfluidic flow block where blank buffer injections can be utilized to eliminate non-bound and loosely bound aptamers to the target-functionalized chip at precisely controlled flow rates, while monitoring the binding events in real time [49].

Recent advancements in SELEX technologies are geared towards the automation of repetitive processes for quick process turnaround and high reproducibility, miniaturization of instrumentation to improve efficiency and scalability, and incorporation of emerging technologies in functionalization, sequencing, chemical synthesis, and characterization. Some of these innovative SELEX technologies involve robotic SELEX, microfluidic SELEX, and next-generation sequencing-coupled SELEX [4,50,51]. Additionally, Wu et al. recently reported an advanced SELEX workflow, incorporating multiparametric fluorescence-activated cell sorting to rapidly, quantitatively identify individual aptamers with high affinity and selectivity for targets following a liquid-phase incubation period. The authors also described a click-chemistry approach to aptamer base modifications, generating non-natural aptamers and opening new aptamer possibilities beyond sequences obtainable from natural oligonucleotide libraries [52]. In another recent development, capillary electrophoresis SELEX was implemented by simultaneously incubating two proteins, generating competing migration pressures and high-resolution elution, significantly reducing the number of cycles required to obtain high-affinity aptamers [53]. Many such instances have been reported in the literature where technological advancements in organic chemistry, materials science, bioinformatics, and other domains are incorporated to improve the speed, efficiency, and range of SELEX techniques. These have resulted in optimized cell SELEX, in silico or computer-assisted SELEX, electrochemical SELEX, hydrodynamic SELEX, and many other such innovative techniques for aptamer production [50,54,55,56].

The rapid growth of the aptamer industry is attributable to aptamers’ functional features that give them immense advantages over alternatives, like antibodies, which are largely mainstay affinity probes in biosensor design [57]. Generally, aptamers are much smaller molecules than antibodies and their derivatives. They are therefore less prone to immunogenicity and hepatotoxicity and have good circulation profiles, driving extensive research on their potential therapeutic and in vivo exploratory applications [6]. Their small size also makes them able to access epitopes on biomolecules that are inaccessible to antibodies due to steric hindrance, providing more efficacious binding events [4]. Aptamers are relatively stable in various non-physiological chemical and thermal conditions that would render antibodies unusable. Structural deformations of aptamers are also generally reversible, and they can be easily reused in multiple biosensing cycles [58]. The production of aptamers is typically by chemical synthesis in scalable and economically efficient workflows that circumvent the laboriousness, risk of contamination, time and resource intensiveness, animal sacrifice, and batch-to-batch variability in antibody production. The tunability of aptamer production and stability of the entities make the processes exciting playgrounds for organic chemists who can easily modify the aptamers with tags and labels, without compromising their binding properties, as is often encountered with antibodies [8]. Perhaps most importantly, antibody probes are designed based on highly understood physiological or immunological events that involve the biomarkers of interest, while the demonstrated versatility of aptamers and heavily experimental approach to their production make it possible to produce aptamer probes for less-understood biomarkers, to which the aptamers bind through highly flexible 3D conformations, earning them the name molecular origami. Due to these advantageous features, aptamers are projected to usurp antibody affinity probes, especially in biosensor design, despite the latter’s long-reigning ubiquity in biomedicine [58].

### 3.2. Aptamer Surface Immobilization Strategies

**Figure 3:**
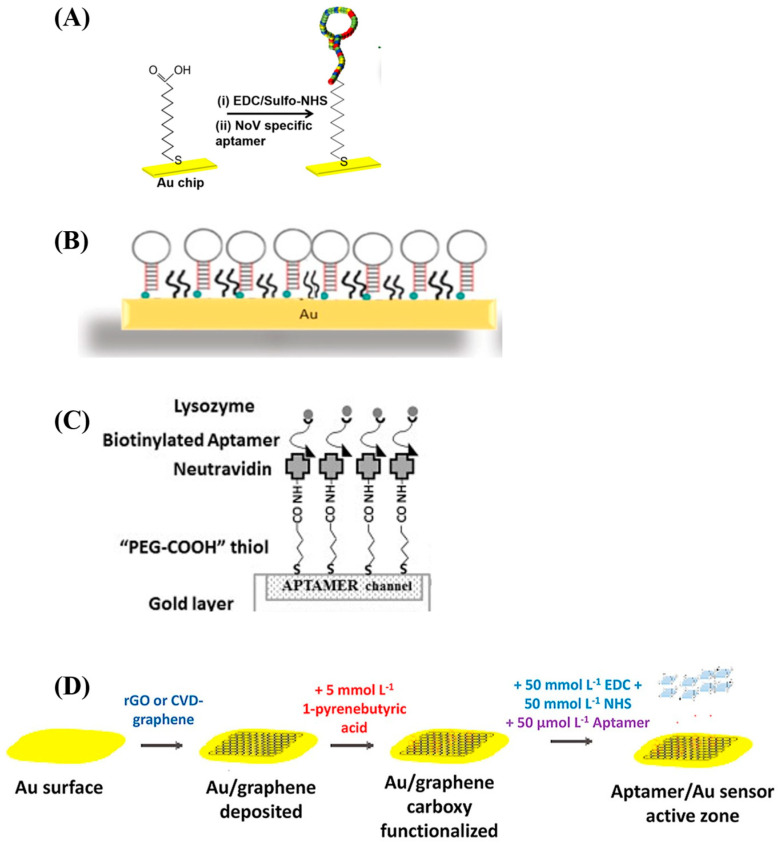
Illustrations of various SPR aptasensor surface preparation strategies. (**A**) The immobilization of a Norovirus capsid aptamer molecule on an SPR chip by amine coupling. The sensor chip is modified with mercaptoundecanoic acid (MUA) which is anchored to the gold film by thiol–gold (S—Au) covalent bonding. The carboxylic acid group of the MUA molecule is activated by EDC/NHS, after which the 5’amine aptamer is reacted with the activated carboxylate. Reproduced with permission from ref. [65]. Copyright 2018, Elsevier B.V. (**B**) A whole-cell *Staphylococcus aureus* aptamer immobilized on an SPR chip by thiol coupling. Adapted with permission from ref. [23]. Copyright 2020 American Chemical Society. (**C**) A lysozyme aptamer immobilized by high-affinity capture. Thiolated PEG-COOH is self-assembled on the chip surface, after which neutravidin is coated on the PEGylated surface by amine coupling. The biotinylated aptamers are then captured through high-affinity neutravidin–biotin capture. Reused with permission from ref. [66]. Copyright 2014 Elsevier B.V. (**D**) A multi-step procedure for the preparation of an SPR aptasensor surface. Graphene is deposited on the gold surface by chemical vapor deposition, after which PBA is π-stacked and its carboxylic groups are activated for the amine coupling of the NH_2_-functionalized aptamers. Reproduced with permission from ref. [60]. Copyright 2021 Elsevier B.V.

Aptamers have been incorporated in SPR biosensor setups, yielding SPR aptasensors that have been used both for the detection of target analytes and to characterize aptameric binding to target molecules. Various covalent and non-covalent methods have been explored to functionalize SPR sensor chip surfaces with aptamers in rational aptamer design-led approaches. Some of the surface preparation methods reported in the literature include amine coupling, π-stacking, and affinity capture, while some studies combine multiple techniques to achieve stable aptasensor surfaces [59]. In an SPR aptasensor designed for the detection of kanamycin residues in food for instance, Écija-Arenas et al. functionalized a gold surface with graphene by chemical vapor deposition, after which pyrene butyric acid was attached to the surface by π-stacking, and the carboxylic acid group-rich residue was activated by a carbodiimide–succinimide reaction that generated covalent bonds with amine groups terminally attached to the aptamers [60]. Conversely, Song et al. functionalized a streptavidin-coated gold chip through the affinity capture of biotinylated DNA aptamers [61]. The high tunability of aptamer synthesis and ease of chemical modifications renders them capable of functionalization with groups and moieties that make precisely designed and site-oriented immobilization easy. Aforementioned are only two of several other examples of SPR aptasensor surface preparation strategies, some of which are presented in Table 1. General workflows of various immobilization techniques used in aptasensor surface preparation are also illustrated in Figure 3.

The immobilization strategy employed in aptasensor surface preparation must be optimized, due to its important ramifications for aptamer binding properties and the performance of biosensors. Non-specific interactions between an aptamer and sensor surface, for example, the excessive proximity of the aptamer binding region to the sensor surface, or steric hindrance arising from high aptasensor surface density may impair access to the binding site, reducing binding affinity [62]. Peculiarly in SPR systems, recent studies have demonstrated that conformational changes in aptamer molecules following target binding further enhance the plasmonic response, leading to the phenomenon of the non-additivity of refractive index increment [63]. Importantly, the overcrowding of the aptasensor surface may prevent these conformational changes, negatively affecting the signal strength and complex stability, with consequences for sensor sensitivity and performance [62]. To better control aptasensor surface density and aptamer orientation and reduce the effects of artifacts arising from the sensor surface, linker molecules are used, including nucleotide sequences [64] and avidin–biotin tags. While these strategies are routinely employed in the literature, there are unfortunately only few studies that properly characterize and report optimization strategies for the surface density of their aptasensors.

**Table 1:** Examples of SPR aptasensor surface preparation strategies.

Analyte of Interest	5′-Aptamer Variable Sequence-3′	Aptamer Preparation Method	Sensor Surface Preparation	Ref.
Whole Avian Influenza Virus—H5Nx	-CCC AGG TCG TGG TGG GTA CTG CGT ATG TGC- -TAG CCC CAG GCG GTG CGA GCT ACT GCC ATT GCAC- -TAA CGG TGT GGC CCG GGG GTA CAG CGC ACT- -TAC AAG TTG GAG GGG TTA AAT GTC TGC CGC- -GGC ATC GTT GGT TAA CCT CAT CAC GCG GGC- -TAA ATG GGC GTG GGA ATG ACT CTA CGG GGC-	Graphene oxide-assisted SELEX (GO-SELEX) and negative SELEX	Streptavidin captures biotinylated aptamers. Di-thiopropoinic acid (DTP) self-assembled monolayers are formed on gold film surface and carboxylic acid groups are activated via EDC/NHS reaction to covalently bind streptavidin which is used to capture biotinylated aptamers.	[67]
Visceral Adipose Tissue-Derived Serpin (vaspin)	-TGA TGG TGT GGC GGG GGG GGC CTG GGG GGG GCC GCC GAT G-	GO-SELEX	DTP self-assembly, streptavidin amine coupling, and capture of 5’T_10_-biotinylated aptamer.	[68]
Norovirus Capsid Protein	- -GCT AGC GAA TTC CGT ACG AAG GGC GAA TTC CAC ATT GGG CTG CAG CCC GGG GGA TCC- -GTC TGT AGT AGG GAG GAT GGT CCG GGG CCC CGA GAC GAC GTT ATC AGG C- -CGT ACG GAA TTC GCT AGC ACG GGG CTT AAG GAA TAC AGA TGT ACT ACC GAG CTC ATG AGG ATC CGA GCT CCA CGT G- -CGT ACG GAA TTC GCT AGC CGA CGG TCA ATG CTC GTG AGC CAG TAC ACA CAA TAT ATG TGG ATC CGA GCT CCA CGT G-	Ordinary SELEX with next-generation sequencing	Mercaptoundecanoic acid (MUA) self-assembly and onward amine coupling of 5’-amine-modified aptamers.	[65]
*Salmonella typhimurium* Outer Membrane Proteins	-TAT GGC GGC GTC ACC CGA CGG GGA CTT GAC ATT ATG ACA G- -GAG GAA AGT CTA TAG CAG AGG AGA TGT GTG AAC CGA GTA A-	Magnetic bead SELEX	Cysteamine self-assembly, carboxymethylated dextran coating, and amine-coupling of amine-modified aptamers.	[69]
*Salmonella typhimurium*	-TAT GGC GGC GTC ACC CGA CGG GGA CTT GAC ATT ATG ACA G-	-	Self-assembly of thiolated aptamer molecules on gold nanoparticles.	[24]
Ochratoxin, Aflatoxin, Adenosine Triphosphate, and Potassium Ions.	-TTT TTG TGG GTA GGG GGG GTT GGA CCA CAC CAA CC- -TTT TTA ACC TGG GGG AGT ATT GCG GAG GAA GGT- -TTT TTG TTG GGC ACG TGT TGT CTC TCT GTG TCT CGT GCC CTT CGC TAG GCC CAC A- -TTT TTG ATC GGG TGT GGG TGG CGT AAA GGG AGC ATC GGA CA-	-	Self-assembly of thiolated aptamers on gold nanorods.	[25]
25-Hydroxyvitamin D3	-AGC AGC ACA GAG GTC ATG GGG GGT GTG ACT TTG GTG TGC CTA TGC GTG CTA CGG AA-	GO-SELEX	Self-assembly of thiolated aptamers on gold nanorods.	[26]
Whole *Staphylococcus aureus* Cells	-TCC CAC GAT CTC ATT AGT CTG TGG ATA AGC GTG GGA CGT CTA TGA-	Whole-cell SELEX	Self-assembly of thiolated aptamers, following reduction of disulfide bonds in oligonucleotide molecules.	[23]
Ochratoxin	-TTT TTG ATC GGG TGT GGG TGG CGT AAA GGG AGC ATC GGA CA-	-	Self-assembly of thiolated aptamers on gold nanorods.	[70]
Whole Shigella Cells	-TTT TTT TTT TTT AGT CTT TCG CTG TTG CTG CTG ATG CC- -Cy5.5-GGC ATC AGC AGC AAC AGC GAA AGA CT-	Whole-cell SELEX	Streptavidin capture of biotinylated aptamer fragment.	[71]
Lysozyme Allergen	-GGG AAT GGA TCC ACA TCT ACG AAT TCA TCA GGG CTA AAG AG-	Robotic SELEX	Neutravidin capture of biotinylated aptamer.	[66]
HER2	-TCT AAA AGG ATT CTT CCC AAG GGG ATC CAA TTC AAA CAG-	Adherent whole-cell SELEX with next-generation sequencing	Self-assembly of thiol-modified aptamers on gold-coated optical fiber surface.	[72]
MCF-7 Breast Cancer Cells	-GCA GTT GAT CCT TTG GAT ACC CTG G-	-	Thiol coupling of aptamer cysteine group to gold surface.	[73]
Tyrosine Kinase-7	-ATC TAA CTG CTG CGC CGC CGG GAA AAT ACT GTA CGG TTA GAT TTT TTT TTT-	-	Thiol coupling of aptamers to gold nanostar surface.	[74]

## 4. Translational Perspective of SPR Aptasensors

### 4.1. Drug Discovery Applications

SPR biosensors have generally been integrated into the drug discovery and development process in the pharmaceutical industry. Broadly, SPR biosensors offer avenues to profile the kinetics of biomolecular interactions towards lead generation, optimization, high-throughput screening, and comparative studies in biosimilarity evaluation. One of the crucial steps in SPR-led drug development is the reproducible site-oriented immobilization or capture of biologic agents for the onward characterization of their interactions with targets. To illustrate, proteins A and G, as well as modified Fc-receptors, are examples of capture molecules that have been applied for the Fc-capture of immunoglobulins, for example [36,75]. While these methods have achieved widespread use in this regard, significant variations exist in their affinity for different classes of investigational biologics, essentially narrowing the range of drug candidates for which stable immobilization can be achieved and negatively impacting drug development.

In contrast, the exponentially growing war-chest of aptamers make it possible for SPR aptasensors to be applied to a wider range of drugs in development, leveling the drug candidates playing field and eliminating artifacts arising from capture instability that interfere with the binding signals obtained. Additionally, the high affinity of aptamers for small molecules opens up the possibility of applying SPR aptasensors in screening not just biologics but small molecules as well, based on highly reproducible drug–target characterization. At the moment, there have been some safe instances in the literature where SPR aptasensors have been applied for drug development like in an anti-cancer bio-barcode drug screening assays [76], SPR aptasensors have yet to be significantly utilized in drug development as capture molecules. However, their functional peculiarities, reproducibility, and ease of production make it reasonable to expect aptamers to be repurposed for highly stable and reversible drug capture for kinetic screening in the near future.

### 4.2. Diagnostics and Disease Monitoring

In contrast to drug development applications, SPR aptasensors have found more extensive use in diagnostic and disease monitoring applications, leveraging highly specific interactions of sensor surface-immobilized aptamers with target biomarker molecules. The relatively small sizes of aptamers ensures that they can be immobilized on the sensor surface at well-controlled surface densities; this is especially important for high-molecular-weight analytes to minimize steric hindrance and electrostatic artifacts and maximize predictability of the aptasensor’s maximum binding capacity [60]. The higher analyte-probe mass ratio that develops as a consequence of the small sizes of aptamers also offers a competitive edge, especially as SPR biosensors rely on mass-induced signal transduction mechanisms [77]. SPR aptasensors have been designed for biosensing purposes in two major modes; direct binding and sandwich detection modes [78]. Direct binding detection aptasensor designs characterize binding events between unit immobilized aptamer molecules to single target molecules, applying simple one-to-one binding models for signal transduction and processing. Direct binding is frequently sufficient for SPR aptasensor-based detection and is especially appropriate for small molecules with only one accessible binding epitope. Additionally, direct-binding biosensing is quick, requires only a few experimental steps, and can be analyzed with simple binding models [78]. The direct-binding or one-site SPR aptasensor biosensing approach is widely reported in the literature for the detection of various biomarkers and whole-cell-pathogenic organisms [79,80,81,82]. Conversely, sandwich detection modes of SPR aptasensors are designed to amplify the SPR signal in the detection of target molecules with multiple accessible binding epitopes. In a workflow similar to other sandwich immunoassays, sandwich SPR aptasensor configurations primarily involve target molecule binding by the immobilized aptamers and the subsequent binding of the captured target molecules by a detection aptamer, conjugated with a signal amplification tag like a plasmonic nanomaterial, a mass tag, or an enzyme molecule [83,84]. Some other innovative signal amplification sandwich assays have been developed that leverage the binding-induced refolding of aptamers and assembly with signal amplifying tags [25]. Creative assay designs like this underscore an additional advantage of SPR aptasensors compared to antibody-based SPR biosensors, due to aptamer flexibility and structural features.

The applications of SPR aptasensors traverse various biomedical fields and have been developed to detect disease biomarkers, pathogens, and small molecules for diagnosis, disease monitoring, and food/agricultural quality control purposes. A recent review of SPR aptasensors in viral disease diagnoses, for example, highlights whole viruses, viral proteins, pseudo-viral materials, and viral biomolecules like enzymes as target molecules of SPR aptasensors reported in the literature for the diagnosis of viral diseases like SARS-CoV2, HIV, the Crimean Congo Hemorrhagic Fever virus, norovirus, dengue virus, and Mpox virus [85]. Similarly, SPR aptasensors have been extensively adopted in food quality monitoring, for detecting small molecules like penicillin and aminoglycoside antibiotics; pathogenic microorganisms like salmonella and streptococci; and toxins like aflatoxins and mycotoxins [60,86]. In disease biomarker detection, SPR aptasensors have been reported to detect and quantify cancer biomarkers like receptor tyrosine protein kinase (HER2), circulating tumor cells (CTC), and circulating tumor DNA (ctDNA), which have been established as reliable markers of progression in different cancer types [87]. The translational potential of SPR aptasensors in point-of-care testing, though not fully explored yet, is apparent in compact innovative SPR aptasensor designs demonstrably functionalized for the rapid detection and high-throughput screening of key biomarkers like toxins, pathogens, and disease biomarkers [88,89].

Comparatively, SPR aptasensors are better suited for translational applications that require in-depth studies of binding dynamics between chemical and biochemical species. In clinical applications especially, SPR biosensors generally report lower sensitivities compared to gold standards like ELISA, limiting their applications in these contexts. However, they are advantageous in the rich information they can provide on biochemical complex formation and stability [90]. This could explain why SPR biosensors are more widely used in industrial applications and for drug development where detailed binding kinetic information enables better drug candidate optimization, while relatively fewer applications have been reported in clinical diagnostics.

**Figure 4:**
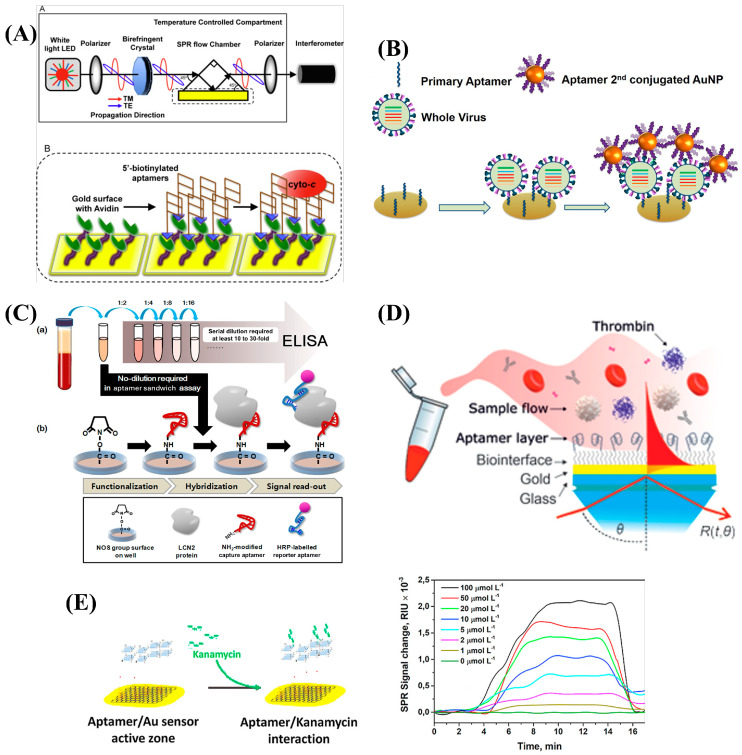
Some examples of SPR aptasensor applications in drug screening and infectious pathogen, disease biomarker, and small-molecule detection. (**A**) An SPR aptasensor for anti-cancer drug screening. Reproduced with permission from ref. [91]. Copyright 2014, Elsevier B.V. (**B**) A dual-aptamer SPR biosensor of whole avian flu virus with gold nanoparticle conjugation for signal amplification. Reproduced with permission from ref. [67]. Copyright 2016 Elsevier B.V. (**C**) A sandwich detection-based SPR aptasensor designed for sandwich immunoassay for Hepatocellular Carcinoma diagnosis [92]. (**D**) An SPR aptasensor for thrombin detection in human blood. Reproduced with permission from ref. [80]. Copyright 2020, Elsevier B.V. (**E**) Kanamycin residues detection in food by an SPR aptasensor. Reproduced with permission from ref. [60]. Copyright 2021 Elsevier B.V.

Some examples of SPR aptasensor setups are illustrated in Figure 4, while a list of SPR aptasensors including their affinity, configuration, and sensitivities is provided in Table 2.

### 4.3. Regulatory Considerations and Broader Impact

Regulatory frameworks for biosensors’ clinical approvals and manufacturing are decentralized across global regions, with major regulatory bodies and frameworks like the US Food and Drug Agency (FDA), Chinese State Administration for Market Regulation (SAMR), and EU In Vitro Diagnostic Medical Device Directive (IVDMDD) providing regional oversight and direction [93]. These frameworks align in their general handling of biosensors as medical devices, which the 21st Title of the Code of Federal Regulations regards as “reagents, instruments, and systems intended for use in the diagnosis of disease or in the determination of the state of health to cure, mitigate, treat, or prevent disease” as upheld by the US FDA [94]. Accordingly, developing biosensors for clinical purposes must generally satisfy strict stipulations of their documented intended use, analytical validation, and clinical performance quality attributes. This is common to the frameworks developed by major regional regulatory bodies globally. In essence, a biosensor typically progresses through a comprehensive documentation of the target analyte it is touted to detect and/or quantify, as well as its intended usage circumstances, and through lab-scale analytical tests that ascertain the sensitivity, specificity, accuracy, precision, robustness, and reproducibility of biosensing, after which clinical validation is conducted to demonstrate the clinical value of the biosensor [94,95].

Different biosensors have been put through this rigorous procedure to ensure that their translational deployment does not constitute clinical and/or public health detriments. Aptamers are a recent addition to the biomedical science toolbox, discovered less than 40 years ago, particularly SPR aptasensors, which are largely in proof-of-concept and analytical validation development steps. Some of the challenges with aptamer specificity, the reproducibility of findings, and the rigorousness of testing required to transition aptasensors through technology readiness levels, coupled with the nascence of the field, may explain the dearth of SPR aptasensors in widespread clinical diagnostics and industrial adoption [58].

Regulatory bureaucracies notwithstanding, the translational potential of SPR aptasensors is undeniable, as is their imminent broader impact at the socioeconomic and healthcare accessibility levels. As has been repeatedly demonstrated in regional and global public health incidents like the Ebola virus and COVID-19, there remains inequity in access to diagnostic services, both on the international level and within regions with marked socioeconomic stratifications and inadequate health systems [96]. These inequalities result from an interplay of geopolitical circumstances and, crucially, the realities of costs associated with developing diagnostic technology that scales through the hurdles to translational deployment; training personnel to use, maintain, and advance the technology; the availability of data for post-marketing surveillance; and the adjacent demands of state-of-the-art diagnostic technology. Much remains to be performed to mitigate these challenges and ensure healthcare accessibility across the socioeconomic spectrum. However, the low costs, scalability, and reproducibility of SELEX for aptamer production, their integration with widely existing technologies, and the development of a relatively low-cost, miniaturized SPR setup promise to democratize access to SPR aptasensors for accessible biosensing [97].

Sustainability is one of the most important considerations in the discourse over the translation of SPR aptasensors to clinical and industrial scale use. As SPR aptasensors ascend technology readiness levels, they must demonstrate an alignment with the overarching goals of sustainability through environmentally safe materials and processes. Generally, SPR aptasensors already provide opportunities for advanced biosensing in the agricultural, food, and environmental monitoring industries [98]. Beyond using them as innovative tools to address environmental concerns, there is also a need to pursue mainstream decarbonization and the general eco-friendliness of SPR aptasensor manufacturing processes. On the one hand, using materials from renewable sources to design and fabricate SPR aptasensors provides a promising avenue. An example is the use of graphene and graphitic materials in sensor surface preparation, which can be reproducibly synthesized from recycled or upcycled waste resources [99,100]. While this is a relatively under-explored discourse as far as SPR aptasensors are concerned, fully realizing their translational potential may require that the sustainability of their development and deployment is thoroughly established through extensive life cycle analyses, the prioritization of renewable sources of component materials, green chemistry in sensor preparation and/or functionalization, sustainable manufacturing processes, and strategies for environmentally friendly end-of-life handling [101].

Several aptasensors have been commercialized, including AptameX, a significantly successful colorimetric aptasensor developed for the diagnosis of COVID-19 [102]. Additionally, there are various ongoing clinical trials of aptasensors developed for medication therapy monitoring, oncologic conditions, and reproductive health, as well as approved patents for techniques in producing various aptasensors. These, alongside the commercial success of aptamer-based therapeutics, underscore their translational potential, which may soon result in an uptrend in plasmonic aptasensor commercialization [8]. Some commercialized, patented, and clinically tested aptasensors are presented in Table 3.

## 5. Challenges and Limitations of SPR Aptasensors

Despite the multi-dimensional potential of SPR aptasensors, there are limitations still to be addressed in their design and deployment landscape, emanating from biophysical and analytical challenges associated with aptamers; the availability of established workflows for the at-scale production of SPR aptasensors; and the long-term stability and reusability of SPR aptasensors. The selection of aptamers is a largely combinatorial experimental workflow; relatively little theoretical understanding precedes the process, introducing significant blind spots. The specificity of aptamers, for example, is often improved through counter-selection SELEX steps, the efficacy of which is debated, as aptamers are still widely reported to demonstrate non-specific and promiscuous binding, significantly hampering their commercialization in diagnostics and therapeutics [109]. About a thousand unique aptamers have been selected to date; some inexplicably demonstrate higher specificity and affinity and form more stable complexes with their targets than others [58]. Important variables that significantly affect aptamer–target binding events have yet to be understood.

Some of the most widely cited aptamers have been subjected to extensive experimental analyses and post-selection modification that provides additional control over their binding properties. In contrast, there are still debates over whether certain other aptamers bind the targets they have been reported to bind [58,117]. This heterogeneity in the aptamer landscape is a consequence of the relative novelty of the field. It necessitates rigorous characterization of existing and emerging aptamers and theoretical explorations of the factors governing their activities before they can reliably be integrated into SPR biosensor setups at scale. There is also a drawback in aptamer stability due to matrix effects in complex samples, significantly affecting aptamer stability and performance. A large knowledge gap exists in aptamer characterization and theoretical understanding, even as new aptamers are churned out at high rates; this needs to be addressed for better aptasensor quality assurance [97]. Some recent studies have attempted to improve aptamer sensitivity, specificity, and performance through serum-assisted SELEX and multivalent aptamers [118,119]. Some innovative techniques have also emerged to achieve higher functional aptamer performance, including computational aptamer design, the introduction of physical forces such as magnetic field or electro-hydrodynamics during SELEX, and non-SELEX methods which circumvent PCR amplifications of aptamer motifs [120]. Additionally, innovative aptasensor designs combine multiple biorecognition elements and/or signal transduction modalities to achieve better specificity, sensitivity, and clinical performance in plasmonic aptasensors in complex biological samples [73].

Another limitation of SPR aptasensors that must be surmounted for the technology to proceed towards translational use is the establishment of simple, reproducible, and scalable manufacturing processes. Notably, SPR aptasensors require the meticulous optimization of experimental conditions during fabrication and utilization to achieve acceptable sensitivity and selectivity. This has largely stifled SPR aptasensor development, with other simpler and more predictable signal transduction modalities pursued in aptasensor research instead [77]. This challenge is expected to persist as SPR aptasensors transition to large-scale manufacturing, where batch variability, process parameterization, and conformance to critical quality attributes are key for success. Recent attempts to address the scalable fabrication of aptasensors include a recent comprehensive process parameterization of an electrochemical aptasensor fabrication, using waste rice husk-derived silica nanoparticles [121]. This and similar studies that combine computational analyses with considerations of raw material source and process optimization promise to advance the translational impact of aptasensors generally and SPR aptasensors in particular. Using bio-derived nanomaterials for sensor chips could reduce the environmental footprint of mass-produced SPR aptasensors.

There is also a paucity of information on the long-term stability and reusability of SPR aptasensors in the literature. Expectedly, aptasensors are widely believed to be superior to antibody-based biosensors in terms of longevity and reusability. This is based on the relative stability of aptamers and reversibility of their structural deformations. In essence, while antibodies suffer irreversible and progressive structural damage even during normal use for biosensing purposes, aptasensors are expected to be reusable and durable since the biorecognition elements—aptamers—can withstand non-physiological storage conditions, and because in the right conditions, they can revert to their initial tertiary conformations after a binding and dissociation event [122]. However, translational applications of aptasensors would require them to be subjected to long-term stability studies, as well as accelerated studies to properly obtain an understanding of their long-term potency as biorecognition elements and the effects of several storage, handling, and logistical conditions on their activities beyond the laboratory setting.

## 6. Conclusions and Future Directions

SPR aptasensors combine the mechanistic versatility of SPR biosensors with the functional advantages of aptamers as biorecognition elements. From a translational perspective, this is a new, emerging biosensor field with immense potential in healthcare, environmental, agricultural, and industrial applications. The major advantage of SPR aptasensors derives from the relatively small size of aptamers and their implications for SPR biosensing, being a mass-dependent signal transduction system, as well as the low production costs, simple workflow, process tunability, and ease of functionalization for optimal immobilization and signal amplification. This review has examined the theoretical principles underlying the SPR phenomenon as a biosensing tool and how aptamers are key in this mechanism as a biorecognition element. We have also discussed the SELEX workflow for aptamer production and the applications of aptamer-functionalized SPR biosensors in drug development and diagnostics. Notably, the discourse on SPR aptasensors in the literature is sparse regarding the translational perspective, necessitating our discussion of the major considerations, such as the regulatory framework, broader impact, and sustainability concerns of SPR aptasensors.

The SPR aptasensor field is growing, and several challenges must be surmounted to achieve translational impact. Some of these key challenges involve limited theoretical understanding of aptamer binding behaviors and their implications for characterization, which could limit their adoption rate in biosensing setups. Additionally, simple, reproducible, and scalable fabrication processes and the long-term quality profiling of SPR aptasensors are a definite requirement in the transition to large-scale deployment. There are already attempts to address these limitations in the current literature, and it is expected that the field of SPR aptasensors will experience new research approaches in the coming years that center on the most crucial limitations to translational adoption in their experimental design. Additionally, theoretical and computational models of aptamer–target complexes, which are already in development, will expectedly continue to emerge towards enriching the scientific understanding of principles that govern these interactions, especially as aptamers have recently been found within in vivo systems, suggesting a physiological role [123].

Emerging aptamer engineering approaches suggest further diversification of the aptamer pool and rational design of aptamers with exciting prospects in biosensing applications. For example, the introduction of non-natural analogs and the chemical modification of nucleotide bases have been touted as strategies to chemically diversify aptamers and introduce control over binding activity, while fortifying them with nuclease resistance for in situ applications [2]. Additionally, signal amplification strategies that do not compromise analyte samples are being pursued through dual-aptamer recognition strategies in various symmetric or asymmetric configurations, including in conjunction with antibodies whose larger molecular weights provide advantages as detection probes [2].

Some advanced technologies that introduce multimodality in SPR aptasensor design and artificial intelligence- or machine learning-led data processing are exciting developments to watch soon; they could significantly shape the future outlook of SPR aptasensors, especially as it pertains to their translational deployment in complex, unpredictable systems. Artificial intelligence and machine learning have been applied to improve aptasensor surface preparation for optimal performance and in signal processing for high-throughput screening and streamlined decision making in complex, multivariate circumstances. This has produced innovative plasmonic and optical aptasensors for SARS-CoV2 detection [124], neurodegenerative and infectious diseases diagnoses [125,126], and in environmental monitoring [127].

Combining the tunability, stability, versatility, and ease of fabrication of aptamers with the real-time, label-free modality of SPR biosensors, the potential of SPR aptasensors and other plasmonic aptasensors is immense. Recent advancements in next-generation, high-performance materials, sustainable approaches, and exponentially growing computational capacity are set to pave the way for the mainstream adoption of SPR aptasensors.

## Figures and Tables

**Table 2:** Examples of SPR aptasensors in the literature.

Analyte of Interest	5′-Aptamer Sequence-3′	Affinity Values	SPR Configuration	Signal Amplification	Limit of Detection	Ref.
Visceral Adipose tissue-Derived Serpin (vaspin)	-TGA TGG TGT GGC GGG GGG GGC CTG GGG GGG GCC GCC GAT G-	K_D_: 0.17–0.24 µM	Prism-coupled SPR	Sandwich SPR with gold nanoparticle-conjugated secondary aptamer	3.5–4.7 ng/mL	[68]
Norovirus Capsid Protein	-GCT AGC GAA TTC CGT ACG AAG GGC GAA TTC CAC ATT GGG CTG CAG CCC GGG GGA TCC- -GTC TGT AGT AGG GAG GAT GGT CCG GGG CCC CGA GAC GAC GTT ATC AGG C- -CGT ACG GAA TTC GCT AGC ACG GGG CTT AAG GAA TAC AGA TGT ACT ACC GAG CTC ATG AGG ATC CGA GCT CCA CGT G- -CGT ACG GAA TTC GCT AGC CGA CGG TCA ATG CTC GTG AGC CAG TAC ACA CAA TAT ATG TGG ATC CGA GCT CCA CGT G-	K_D_: 1.73–21.4 nM	Prism-coupled SPR	Sandwich SPR with gold nanorod-conjugated detection aptamers	70 aM	[65]
*Salmonella typhimurium* Outer Membrane Proteins	-TAT GGC GGC GTC ACC CGA CGG GGA CTT GAC ATT ATG ACA G- -GAG GAA AGT CTA TAG CAG AGG AGA TGT GTG AAC CGA GTA A-	K_off_: 5.2 × 10^−3^–7.4 × 10^−3^ s^−1^	Prism-coupled SPR	-	3 × 10^4^ CFU mL^−1^.	[69]
*Salmonella typhimurium*	-TAT GGC GGC GTC ACC CGA CGG GGA CTT GAC ATT ATG ACA G-		Glass-coupled LSPR	-	10^4^ CFU/mL	[24]
Ochratoxin, Aflatoxin, Adenosine Triphosphate, and Potassium Ions.	-TTT TTG TGG GTA GGG GGG GTT GGA CCA CAC CAA CC- -TTT TTA ACC TGG GGG AGT ATT GCG GAG GAA GGT- -TTT TTG TTG GGC ACG TGT TGT CTC TCT GTG TCT CGT GCC CTT CGC TAG GCC CAC A- -TTT TTG ATC GGG TGT GGG TGG CGT AAA GGG AGC ATC GGA CA-		Glass-coupled LSPR	LSPR peak shift induction by berberine binding to aptamer–target aptamer G-quadruplex	0.56–1.05 pM	[25]
25-Hydroxyvitamin D3	-AGC AGC ACA GAG GTC ATG GGG GGT GTG ACT TTG GTG TGC CTA TGC GTG CTA CGG AA-	K_D_: 11 nM	Glass-coupled LSPR	-	0.1 ng/mL	[26]
Whole *Staphylococcus aureus* Cells	-TCC CAC GAT CTC ATT AGT CTG TGG ATA AGC GTG GGA CGT CTA TGA-	K_D_: 82.8 nM	Glass-coupled LSPR	-	10^3^ CFU/mL	[23]
Ochratoxin	-TTT TTG ATC GGG TGT GGG TGG CGT AAA GGG AGC ATC GGA CA-		Optical Fiber-based LSPR	-	12 pM	[70]
Whole Shigella Cells	-TTT TTT TTT TTT AGT CTT TCG CTG TTG CTG CTG ATG CC- -Cy5.5—GGC ATC AGC AGC AAC AGC GAA AGA CT-		Multicore Optical Fiber-based LSPR	-	1.56 CFU/mL	[71]
Lysozyme Allergen	-GGG AAT GGA TCC ACA TCT ACG AAT TCA TCA GGG CTA AAG AG-	K_D_: 31–65 nM	Prism-coupled SPR	-	2.4 nM	[66]
HER2	-TCT AAA AGG ATT CTT CCC AAG GGG ATC CAA TTC AAA CAG-	K_D_: 6.2 nM	Optical Fiber-based SPR	Sandwich SPR with anti-HER2 antibodies as detection probes	77.4 pM	[72]
MCF-7 Breast Cancer Cells	-GCA GTT GAT CCT TTG GAT ACC CTG G-		Prism-coupled SPR	Dual recognition together with folic acid-functionalized magnetic nanoparticles for binding overexpressed FA receptor.	500 cells/mL	[73]
Tyrosine Kinase 7 Expressed on Circulating Tumor Cells	-ATC TAA CTG CTG CGC CGC CGG GAA AAT ACT GTA CGG TTA GAT TTT TTT TTT-		Electrochemical LSPR	LSPR-generated hot electrons enhance electrochemical current response	5 cells/mL	[74]
Cytochrome C	-ATC GAT AAG CTT CCA GAG CCG TGT CTG GGG CCG ACC GGC GCA TTG GGT ACG TTG TTG CCG TAG AAT TCC TGC AGC C-		Prism-coupled SPR	Gold nanorods for plasmonic enhancement and RNAse H enzymatic recycling	80 pM	[76]

**Table 3:** Some commercialized, patented, and clinically tested aptamer-based biosensor products and concepts.

tRADE Name/Identifier	Biomarker/Target	Biosensor Setup	Developmental Stage	Ref.
AptoDetect™-Lung	EGFR1, MMP7, CA6, KIT, CRP, C9, and SERPINA3	Proteomic profiling	Commercialized	[103]
Apta-Beacon™	Multiple biomarkers	Colorimetric and fluorometric endpoints	Commercialized	[104]
OLIGOBIND	Thrombin	Fluorometric ELISA	Commercialized	[105]
APTSENS	COVID-19	Electrochemical biosensor	Commercialized	[106]
SOMAscan	Multiple biomarkers	Proteomic profiling	Commercialized	[107]
AflaSense	Aflatoxin	Fluorometry	Commercialized	[108]
OTA-Sense	Ochratoxin A	Sandwich fluorometry	Commercialized	[109]
Apollomer^TM^	Multiple pathogens	Portable electrochemical biosensor	Commercialized	[110]
Patent: CN104634754B	Terramycin	ELISA	Patent active (expected expiration: 2035)	[111]
Patent: US10036073B2	Foodborne pathogens	Fluorometry	Fee-related expired patent (adjusted expiration: 2032)	[112]
Patent: CN113881790A	Foodborne pathogens	PCR and fluorometry	Patent active (expected expiration: 2041)	[113]
Patent: CN103439296B	Adenosine	SPR	Fee-related expired patent (expected expiration: 2033)	[114]
Patent: CN108845009B	Environmental pollutants	Photoelectrochemical sensor	Patent active (expected expiration: 2038)	[115]
Project ADHERE (clinicaltrials.gov ID: NCT04870671)	Tenofovir	Electrochemical aptasensor	Early Phase 1 clinical trials completed.	[116]
AptameX	COVID-19	Colorimetric sensor	Commercialized	[102]
Identify Proteomic Biomarkers for Outcome Prediction of Lipiodol TACE Treatment (Lipiodol TACE)	Multiple biomarkers	Proteomic profiling	Observational clinical trials ongoing	Clinicaltrials.gov ID: NCT04459468
Electro-Phage and Colorimetric Aptamer Sensors for Clinical Staging and Monitoring of Bladder Cancer	Multiple urinary biomarkers	Colorimetric sensor	Observational clinical trials ongoing	Clinicaltrials.gov ID: NCT02957370
Non-Invasive, Highly Specific Detection of Oxytocin in Biological Fluids	Salivary oxytocin	Aptamer-based electrochemical assay	Observational clinical trials completed	Clinicaltrials.gov ID: NCT03140709

## Data Availability

No new data were created or analyzed in this study. Data sharing is not applicable to this article.

## Abbreviations

The following abbreviations are used in this manuscript:

CTC	Circulating tumor cell
ctDNA	Circulating tumor DNA
DNA	Deoxyribonucleic acid
DTP	Di-thiopropionate
EDC	Ethyl di-methyl aminopropyl carbodiimide
FDA	Food and Drug Agency
GO-SELEX	Graphene oxide-based SELEX
HER2	Receptor tyrosine protein kinase
HIV	Human Immunodeficiency Virus
IVDMDD	In Vitro Diagnostic Medical Device Directive
LSPR	Localized surface plasmon resonance
MUA	Mercapto-undecanoic acid
NHS	N-hydroxy succinimide
RNA	Ribonucleic acid
SAMR	Chinese State Administration for Market Regulation
SARS-CoV2	Severe acute respiratory syndrome coronavirus 2
SELEX	Systematic Evolution of Ligands by Exponential Enrichment
SPR	Surface plasmon resonance
SPRi	Surface plasmon resonance imaging
